# Classifying Dementia and Alzheimer's Disease Participants Using Machine Learning Methods Based on Comorbidities and Lifestyle Factors

**DOI:** 10.1002/alz70860_101861

**Published:** 2025-12-23

**Authors:** Jermyn Z See, Kin Jun See, See Ann Soo, Nav Vij, Prem Pillay

**Affiliations:** ^1^ Neurowyzr Pte Ltd, Singapore, Singapore, Singapore; ^2^ Singapore Brain and Spine Specialist Singapore Brain‐Spine‐Nerves Center, Singapore, Singapore, Singapore

## Abstract

**Background:**

Accurate participant classification is crucial in dementia studies for early diagnosis and effective intervention strategies. This study investigates the use of machine learning methods, such as XGBoost, to classify participants with diagnosed dementia, Alzheimer's disease (AD), or mild cognitive impairment (MCI) against a healthy population. By leveraging comorbidities and lifestyle factors as predictive features, this approach also highlights the potential of large datasets like the UK Biobank for future risk prediction in undiagnosed populations.

**Method:**

We analyzed data from more than 500,000 UK Biobank participants. Participants were categorized as healthy, diagnosed dementia, AD, or MCI based on ICD‐10 codes, self‐reported diagnoses, and clinical assessments. Machine learning methods, with initial results based on XGBoost, were employed to classify participants using features such as comorbidities (e.g., hypertension, diabetes) and lifestyle factors (e.g., smoking, physical activity). To address class imbalance, the healthy group was sub‐sampled into five subsets for 5‐fold cross‐validation, and AUROC scores were averaged across all folds and subsets to compute a mean AUROC.

**Result:**

XGBoost demonstrated strong performance in classifying participants based on comorbidities and lifestyle factors, with a mean AUROC across all samples of approximately 0.81. Feature importance analysis revealed that among comorbidities, hypertension, stroke, and heart disease were the most important predictors of cognitively unhealthy participants. For modifiable lifestyle factors, vigorous physical activity, alcohol intake, and smoking emerged as the most influential features. These findings underscore the effectiveness of machine learning methods, with XGBoost providing initial evidence of their utility in distinguishing between healthy individuals and those with diagnosed dementia, AD, or MCI.

**Conclusion:**

This study highlights the potential of machine learning methods, such as XGBoost, to accurately classify participants with diagnosed dementia, Alzheimer's disease, or MCI against a healthy population. By leveraging these features and identifying key predictors through feature importance testing, machine learning methods enhance the precision of participant classification. Additionally, analyzing large publicly available datasets supports the development of future risk prediction models for undiagnosed individuals, ultimately facilitating earlier interventions and improved patient outcomes in dementia care and research.

